# Supplementary material for: Novel Metabolites Associated with Decreased GFR in Finnish Men: A 12-Year Follow-Up of the METSIM Cohort
Source: Int J Mol Sci. 2024 Sep 18;25(18):10044. doi: 10.3390/ijms251810044 (PMC11432478; doi:10.3390/ijms251810044)
Supplement: Supplementary file 1 [file ijms-25-10044-s001.zip › ijms-3198868-supplementary.pdf]

Supplemental information

Supplemental Tables S1 and S2

Table S1. Association of the top 100 metabolites with eGFR in the METSIM study.

Metabolite	T2D			Prediabetes			NGT		
	N	Beta	*p*	N	Beta	*p*	N	Beta	*p*
Creatinine	1057	-0,690	2,4E-214	1057	-0,475	3,2E-84	1055	-0,649	1,5E-167
N,N,N-trimethyl-Alanylproline betaine	1057	-0,586	3,6E-136	1057	-0,423	1,5E-64	1055	-0,489	1,0E-79
N,N-dimethyl-pro-pro	1057	-0,565	3,0E-122	1039	-0,301	1,4E-31	1039	-0,373	1,9E-44
Pseudouridine	1057	-0,551	5,8E-111	1057	-0,401	3,4E-57	1054	-0,421	2,9E-58
Hydroxyasparagine**	1057	-0,561	1,3E-110	1049	-0,336	2,9E-38	1052	-0,416	4,4E-52
1-methylhistidine	1056	-0,533	1,3E-106	1055	-0,396	3,6E-57	1050	-0,453	7,0E-70
N-acetylalanine	1057	-0,535	2,4E-101	1057	-0,347	2,4E-42	1055	-0,415	1,1E-54
N-acetylserine	1057	-0,515	6,1E-95	1057	-0,372	2,7E-49	1054	-0,409	1,4E-53
C-glycosyltryptophan	1057	-0,493	1,3E-83	1057	-0,317	2,3E-35	1055	-0,385	9,5E-47
N6-carbamoylthreonyladenosine	1035	-0,488	1,8E-82	1019	-0,328	1,2E-36	1012	-0,397	2,3E-49
Kynurenate	1057	-0,487	3,3E-79	1057	-0,338	3,0E-39	1055	-0,398	7,6E-48
2,3-dihydroxy-5-methylthio-4-pentenoate	1057	-0,487	7,1E-79	1057	-0,356	5,9E-42	1055	-0,470	4,0E-70
Alpha-ketoglutaramate*	1057	-0,465	6,6E-78	1056	-0,252	7,7E-23	1054	-0,275	1,8E-24
N-acetylthreonine	1056	-0,458	2,3E-73	1057	-0,327	5,6E-38	1053	-0,399	7,9E-53
Erythritol	1056	-0,466	1,5E-71	1055	-0,346	6,4E-39	1052	-0,354	6,3E-38
Erythronate*	1057	-0,443	1,1E-64	1057	-0,292	1,0E-28	1054	-0,315	3,7E-30
N-formylmethionine	1057	-0,430	7,2E-62	1057	-0,326	3,7E-37	1055	-0,385	1,7E-47
Gamma-carboxyglutamate	1055	-0,419	6,2E-60	1029	-0,246	2,3E-21	1032	-0,272	2,7E-23
N-acetylvaline	1057	-0,423	7,4E-60	1057	-0,298	1,9E-31	1055	-0,320	2,9E-32
Sulfate*	1057	-0,410	4,2E-58	1057	-0,240	8,8E-21	1055	-0,254	7,4E-21
Homocitrulline	1054	-0,406	1,4E-54	1017	-0,299	1,9E-29	1033	-0,301	1,2E-28
Urea	1057	-0,402	1,6E-54	1057	-0,240	1,1E-20	1055	-0,266	1,7E-22
(S)-a-amino-omega-Caprolactam	1056	-0,401	7,7E-54	1034	-0,301	4,8E-31	1041	-0,325	1,3E-33
N2,N2-dimethylguanosine	1057	-0,401	4,5E-53	1047	-0,253	2,0E-21	1046	-0,319	2,7E-31
5,6-dihydrouridine	1057	-0,390	2,6E-51	1056	-0,291	5,5E-30	1054	-0,265	1,7E-22
Dimethylarginine (SDMA + ADMA)	1057	-0,390	2,7E-51	1057	-0,222	9,7E-18	1055	-0,285	4,3E-26
Arabonate/xylonate	1056	-0,382	2,9E-48	1056	-0,255	4,1E-23	1054	-0,230	4,2E-17
O-sulfo-L-tyrosine	1057	-0,379	5,2E-48	1057	-0,272	4,1E-26	1055	-0,355	7,7E-41
Picolinoylglycine	1050	-0,392	9,1E-47	1028	-0,289	3,6E-26	1015	-0,356	2,6E-35
Glutarylcarnitine (C5-DC)	1057	-0,363	7,9E-46	1057	-0,257	4,4E-24	1055	-0,255	1,5E-21
2S,3R-dihydroxybutyrate	1057	-0,366	3,5E-45	1057	-0,271	5,3E-27	1054	-0,308	1,9E-30
Indolelactate	1057	-0,362	4,2E-43	1057	-0,301	1,5E-31	1055	-0,347	8,8E-38
4-acetamidobutanoate	1057	-0,357	4,3E-43	1056	-0,253	5,1E-22	1053	-0,240	1,1E-18
Arabitol/xylitol	1056	-0,361	7,7E-43	1055	-0,288	3,7E-29	1054	-0,298	2,5E-28
N-acetylcarnosine	1054	-0,373	1,1E-41	1046	-0,336	2,0E-37	1050	-0,426	6,4E-54
1-methyl-4-imidazoleacetate	1034	-0,353	2,0E-40	1052	-0,273	7,3E-26	1052	-0,331	1,1E-34
Quinolinate	1051	-0,361	3,3E-40	1030	-0,238	1,1E-17	1026	-0,259	1,8E-19

Hydroxy-N6,N6,N6-trimethyllysine*	1052	-0,343	9,5E-40	1055	-0,298	6,7E-31	1054	-0,354	1,8E-40
N6-acetyllysine	1056	-0,348	1,3E-39	1054	-0,214	8,4E-17	1050	-0,246	1,7E-19
1-ribosyl-imidazoleacetate*	1056	-0,349	1,7E-39	1047	-0,241	1,3E-19	1052	-0,289	3,5E-26
N-acetylhistidine	1046	-0,341	1,9E-38	1014	-0,162	1,1E-09	1034	-0,200	1,1E-12
Kynurenine	1057	-0,356	3,8E-38	1057	-0,226	4,5E-17	1055	-0,306	2,4E-27
N1-methyladenosine	1057	-0,331	1,5E-36	1057	-0,206	2,9E-15	1055	-0,249	5,1E-20
3-methylglutaconate	1057	-0,335	3,4E-36	1055	-0,276	2,5E-26	1053	-0,214	7,7E-15
Heptenedioate (C7:1-DC)*	1041	-0,317	1,0E-33	1042	-0,226	3,6E-18	1030	-0,241	1,9E-17
2R,3R-dihydroxybutyrate	1057	-0,320	1,0E-32	1057	-0,182	3,8E-11	1054	-0,253	4,2E-18
3-amino-2-piperidone	1057	-0,310	1,7E-32	1051	-0,184	1,4E-12	1053	-0,190	7,6E-12
Methylsuccinate	1034	-0,319	4,6E-32	1033	-0,184	2,8E-12	1026	-0,229	5,1E-16
3-hydroxy-3-methylglutarate	1057	-0,322	7,8E-32	1056	-0,233	1,4E-18	1050	-0,233	6,7E-17
Methionine sulfone	1057	-0,305	2,1E-31	1040	-0,250	6,9E-22	1045	-0,230	2,3E-17
N-acetyltaurine	1056	-0,306	3,8E-31	1053	-0,202	5,9E-15	1053	-0,185	1,4E-11
Myo-inositol	1057	-0,303	3,9E-31	1055	-0,202	4,4E-15	1054	-0,214	3,7E-15
Xanthurenate	1030	-0,310	6,7E-30	1025	-0,186	3,1E-12	1024	-0,262	1,8E-19
N-acetylmethionine	1056	-0,306	7,5E-30	1057	-0,263	2,4E-24	1055	-0,277	3,1E-24
N-acetylneuraminate	1057	-0,298	9,1E-30	1053	-0,171	5,0E-11	1052	-0,205	5,2E-14
3-methylglutarylcarnitine (2)	1054	-0,303	1,1E-29	1030	-0,203	2,2E-14	1042	-0,200	3,5E-13
N-acetyl-isoputreanine	1057	-0,292	7,9E-29	1055	-0,192	1,1E-13	1055	-0,174	1,6E-10
Gamma-glutamylphenylalanine	1057	-0,300	1,2E-28	1056	-0,260	2,5E-23	1054	-0,254	1,0E-19
tiglylcarnitine (C5:1-DC)	1057	-0,287	2,0E-28	1043	-0,154	2,9E-09	1044	-0,215	2,4E-15
(N(1) + N(8))-acetylspermidine	1057	-0,288	4,6E-28	1055	-0,198	2,8E-14	1054	-0,149	7,5E-08
Pentose acid*	1042	-0,293	2,1E-27	1002	-0,168	3,5E-10	1032	-0,228	3,0E-16
Androsterone glucuronide	1036	-0,289	3,1E-27	1025	-0,179	1,8E-11	1034	-0,249	9,8E-20
1-methyl-5-imidazoleacetate	1036	-0,281	7,0E-26	1043	-0,213	1,4E-16	1051	-0,314	1,1E-31
Glutamine_degradant*	1057	-0,275	3,5E-25	1053	-0,229	1,3E-18	1051	-0,215	3,5E-15
3-hydroxy-2-ethylpropionate	1057	-0,274	3,8E-25	1056	-0,220	1,0E-17	1047	-0,214	6,4E-15
7-methylguanine	1057	-0,276	4,2E-25	1056	-0,257	4,3E-23	1055	-0,263	2,7E-22
Acisoga	1045	-0,270	4,4E-25	1014	-0,137	2,5E-07	1024	-0,104	1,8E-04
5-methylthioadenosine (MTA)	1057	-0,281	6,7E-25	1055	-0,179	1,9E-11	1053	-0,211	9,7E-14
Guaiacol sulfate	1055	-0,273	7,0E-25	1057	-0,213	2,1E-16	1055	-0,258	7,3E-21
Isobutyrylcarnitine (C4)	1057	-0,277	7,3E-25	1049	-0,196	3,6E-14	1051	-0,222	7,7E-16
Adipoylcarnitine (C6-DC)	1057	-0,265	6,2E-24	1042	-0,211	3,3E-16	1049	-0,215	5,4E-15
Etiocholanolone glucuronide	1046	-0,268	1,5E-23	1006	-0,168	3,8E-10	1026	-0,237	1,9E-17
Ribonate	1056	-0,262	6,3E-23	1045	-0,132	3,8E-07	1043	-0,105	1,3E-04
Phenyllactate (PLA)	1057	-0,268	1,3E-22	1057	-0,257	3,7E-23	1055	-0,247	2,0E-19
Trimethylamine N-oxide	1057	-0,255	4,6E-22	1057	-0,160	6,1E-10	1055	-0,204	8,7E-14
N-methylpipecolate	1057	-0,249	2,4E-21	1038	-0,162	6,4E-10	1045	-0,175	1,1E-10
3-aminoisobutyrate	1057	-0,252	8,8E-21	1055	-0,175	3,3E-11	1055	-0,244	5,0E-18

N1-Methyl-2-pyridone-5-carboxamide	1057	-0,248	2,3E-20	1057	-0,159	1,2E-09	1055	-0,247	4,2E-19
3-hydroxyoctanoylcarnitine (1)	1036	-0,249	3,6E-20	1020	-0,183	4,9E-12	1025	-0,238	6,5E-18
2-O-methylascorbic acid	1057	-0,251	5,5E-20	1057	-0,150	3,3E-08	1055	-0,181	1,4E-10
3-formylindole	1057	-0,243	5,7E-20	1057	-0,172	4,5E-11	1052	-0,212	1,9E-14
3-hydroxypyridine sulfate	1055	-0,252	5,9E-20	1051	-0,255	3,0E-23	1053	-0,262	2,0E-21
Deoxycarnitine	1057	-0,239	9,3E-20	1055	-0,243	3,1E-21	1055	-0,232	9,5E-18
Argininate*	1055	-0,241	2,8E-19	1051	-0,147	4,3E-08	1052	-0,182	1,7E-10
6-hydroxyindole sulfate	1054	-0,238	3,7E-19	1056	-0,175	1,9E-11	1053	-0,219	8,0E-16
Glutamine conjugate of C6H10O2 (1)*	1054	-0,245	3,7E-19	1020	-0,166	8,1E-10	1016	-0,160	3,5E-08
3-hydroxydecanoylcarnitine	1036	-0,245	4,6E-19	1013	-0,154	7,9E-09	1022	-0,183	6,6E-11
Imidazole lactate	1051	-0,235	6,3E-19	1051	-0,169	7,1E-11	1054	-0,159	6,7E-09
N6,N6,N6-trimethyllysine	1057	-0,234	9,2E-19	1057	-0,213	2,3E-16	1055	-0,349	4,8E-38
3-methyl catechol sulfate (1)	1053	-0,237	1,3E-18	1054	-0,243	2,8E-21	1052	-0,245	3,0E-19
3-(4-hydroxyphenyl)lactate	1057	-0,242	7,2E-18	1057	-0,213	1,3E-15	1055	-0,201	1,6E-12
Urate	1057	-0,235	7,8E-18	1057	-0,185	3,8E-12	1055	-0,203	9,9E-13
11beta-hydroxyandrosterone glucuronide	1043	-0,233	7,9E-18	1017	-0,147	4,1E-08	1023	-0,195	4,3E-12
undecenoylcarnitine (C11:1)	1057	-0,228	8,8E-18	1055	-0,142	3,5E-08	1055	-0,187	5,2E-12
Citrulline	1057	-0,228	1,0E-17	1057	-0,197	2,5E-14	1055	-0,215	5,7E-15
Ascorbic acid 3-sulfate*	1057	-0,224	1,3E-17	1057	-0,186	4,0E-13	1054	-0,186	6,8E-12
3-indoxyl sulfate	1057	-0,226	1,9E-17	1057	-0,168	1,5E-10	1055	-0,180	4,4E-11
Phenylacetylglutamine	1057	-0,231	4,0E-17	1057	-0,174	4,9E-11	1055	-0,212	5,4E-14
Pyridoxate	1057	-0,222	5,7E-17	1057	-0,145	2,3E-08	1055	-0,232	6,9E-18
3-methylxanthine	1047	-0,219	9,1E-17	1045	-0,178	5,8E-12	1046	-0,224	8,9E-17

Beta and *p* are based on linear regression analyses adjusted for age, BMI, smoking, systolic blood pressure, fasting glucose and total triglycerides. The participants in the groups of type 2 diabetes (T2D), prediabetes and normal glucose tolerance (NGT) were matched for age and BMI. Estimated glomerular filtration rate (eGFR), significant *p* value < 5,0 E10-5.

Table S2. Association of metabolites with eGFR at follow-up in all METSIM participants.

Metabolite		Unadjusted		Model 1		Model 2			Novel
**Amino acids**								**Subclass**	
Hydroxyasparagine**	7066	-0,447	0,00E+00	-0,112	1,20E-33	-0,121	1,10E-37	Alanine and Aspartate Metabolism	No
N-acetylalanine	7082	-0,43	0,00E+00	-0,098	1,90E-26	-0,108	5,10E-32	Alanine and Aspartate Metabolism	No
Creatinine	7082	-0,536	0,00E+00	-0,12	3,80E-31	-0,18	4,30E-60	Creatine Metabolism	No
γ-carboxyglutamate	6929	-0,295	1,10E-138	-0,061	1,40E-12	-0,065	2,60E-14	Glutamate Metabolism	Yes
Alpha-ketoglutaramate*	7071	-0,244	2,50E-96	-0,039	3,60E-06	-0,048	1,40E-08	Glutamate Metabolism	No
N-acetylserine	7079	-0,419	6,00E-299	-0,095	2,20E-25	-0,103	6,20E-30	Glycine, Serine and Threonine Metabolism	No
N-acetylthreonine	7079	-0,368	2,00E-225	-0,076	4,70E-18	-0,089	6,40E-24	Glycine, Serine and Threonine Metabolism	No
Glycine	7082	-0,126	1,50E-26	-0,05	5,10E-10	-0,056	3,30E-11	Glycine, Serine and Threonine Metabolism	No
Guanidinosuccinate	4685	-0,274	3,20E-81	-0,082	4,00E-16	-0,09	3,60E-18	Guanidino and Acetamido Metabolism	No
4-guanidinobutanoate	7049	-0,158	1,70E-40	-0,048	5,40E-09	-0,049	2,30E-09	Guanidino and Acetamido Metabolism	Yes
1-ribosyl-imidazoleacetate*	7068	-0,317	3,10E-164	-0,087	1,20E-24	-0,09	1,00E-25	Histidine Metabolism	No
N-acetylcarnosine	7059	-0,267	2,80E-115	-0,042	9,70E-07	-0,078	8,90E-18	Histidine Metabolism	No
1-methylhistidine	7048	-0,382	2,90E-244	-0,054	2,20E-09	-0,078	2,90E-17	Histidine Metabolism	No
1-methyl-4-imidazoleacetate	7054	-0,295	3,30E-141	-0,064	3,80E-14	-0,065	3,00E-14	Histidine Metabolism	No
1-methyl-5-imidazoleacetate	7034	-0,212	1,30E-72	-0,037	1,10E-05	-0,047	1,70E-08	Histidine Metabolism	No
Hydantoin-5-propionate	6154	-0,211	3,60E-63	-0,042	1,80E-06	-0,043	1,10E-06	Histidine Metabolism	Yes
Imidazole propionate	6877	-0,166	7,00E-44	-0,041	9,00E-07	-0,04	1,00E-06	Histidine Metabolism	No
N-acetylhistidine	6919	-0,214	1,40E-72	-0,039	3,70E-06	-0,037	8,90E-06	Histidine Metabolism	No
N-lactoyl valine	6781	-0,182	2,50E-51	-0,038	7,30E-06	-0,043	3,10E-06	Lactoyl Amino Acid	Yes
N-lactoyl phenylalanine	7033	-0,233	2,70E-87	-0,038	5,70E-06	-0,037	4,40E-05	Lactoyl Amino Acid	Yes

N-acetylvaline	7082	-0,343	1,00E-194	-0,075	3,50E-18	-0,082	2,60E-21	Leucine, Isoleucine and Valine Metabolism	Yes
3-methylglutaconate	7078	-0,292	2,00E-139	-0,074	2,70E-18	-0,069	2,80E-16	Leucine, Isoleucine and Valine Metabolism	No
N-carbamoylvaline	6358	-0,296	1,20E-128	-0,059	3,70E-11	-0,058	7,50E-11	Leucine, Isoleucine and Valine Metabolism	No
3-methylglutarylcarnitine (2)	7001	-0,257	1,10E-105	-0,059	1,70E-12	-0,058	5,80E-12	Leucine, Isoleucine and Valine Metabolism	Yes
Isobutyrylcarnitine (C4)	7065	-0,213	2,10E-73	-0,04	1,40E-06	-0,045	6,30E-08	Leucine, Isoleucine and Valine Metabolism	No
Hydroxy-N6,N6,N6-trimethyllysine*	7060	-0,321	6,40E-169	-0,063	4,40E-13	-0,075	3,00E-18	Lysine Metabolism	No
N6-acetyllysine	7053	-0,259	1,80E-108	-0,039	3,40E-06	-0,042	6,60E-07	Lysine Metabolism	No
N-formylmethionine	7082	-0,399	7,10E-269	-0,095	2,10E-26	-0,097	7,10E-28	Methionine, Cysteine, SAM and Taurine Metabolism	No
2,3-dihydroxy-5-methylthio-4-pentenoate (DMTPA)*	7082	-0,432	0,00E+00	-0,081	3,90E-18	-0,094	1,70E-22	Methionine, Cysteine, SAM and Taurine Metabolism	No
N-acetylmethionine	7080	-0,334	1,40E-183	-0,087	5,30E-24	-0,087	5,50E-24	Methionine, Cysteine, SAM and Taurine Metabolism	Yes
S-adenosylhomocysteine (SAH)	5573	-0,273	1,70E-95	-0,057	1,60E-09	-0,063	2,50E-11	Methionine, Cysteine, SAM and Taurine Metabolism	No
Methionine sulfone	7055	-0,245	7,70E-97	-0,051	9,00E-10	-0,057	1,30E-11	Methionine, Cysteine, SAM and Taurine Metabolism	No
Lanthionine	4582	-0,272	1,00E-78	-0,045	1,40E-05	-0,048	3,20E-06	Methionine, Cysteine, SAM and Taurine Metabolism	No

Cystathionine	6143	-0,213	3,90E-64	-0,045	5,50E-07	-0,042	3,40E-06	Methionine, Cysteine, SAM and Taurine Metabolism	No
N-acetyltaurine	7048	-0,208	1,40E-69	-0,043	2,30E-07	-0,041	7,60E-07	Methionine, Cysteine, SAM and Taurine Metabolism	Yes
4-acetamidobutanoate	7070	-0,307	6,30E-154	-0,065	4,60E-14	-0,063	1,40E-13	Polyamine Metabolism	No
C-glycosyltryptophan	7082	-0,414	4,70E-291	-0,099	3,10E-28	-0,103	5,30E-30	Tryptophan Metabolism	No
Kynurenate	7082	-0,311	1,30E-158	-0,046	1,20E-07	-0,068	1,10E-13	Tryptophan Metabolism	No
Kynurenine	7082	-0,308	1,70E-155	-0,055	1,40E-10	-0,061	3,40E-12	Tryptophan Metabolism	No
Indolepropionate	7002	-0,096	9,20E-16	-0,035	1,50E-05	-0,036	1,30E-05	Tryptophan Metabolism	No
Vanillactate	6119	-0,341	5,80E-166	-0,067	9,80E-13	-0,065	5,10E-12	Tyrosine Metabolism	No
Dopamine 3-O-sulfate	6930	-0,183	2,40E-53	-0,051	7,90E-10	-0,05	1,40E-09	Tyrosine Metabolism	No
Vanillylmandelate (VMA)	6082	-0,322	1,80E-146	-0,056	2,50E-09	-0,046	1,40E-06	Tyrosine Metabolism	No
4-methoxyphenol sulfate	6786	-0,165	1,10E-42	-0,042	4,60E-07	-0,04	2,10E-06	Tyrosine Metabolism	No
N,N,N-trimethyl-alanylproline betaine (TMAP)	7081	-0,439	0,00E+00	-0,085	8,60E-20	-0,112	1,50E-31	Urea cycle; Arginine and Proline Metabolism	No
3-amino-2-piperidone	7074	-0,247	1,10E-98	-0,074	3,40E-19	-0,072	4,30E-18	Urea cycle; Arginine and Proline Metabolism	No
Dimethylarginine (SDMA + ADMA)	7082	-0,303	3,00E-150	-0,063	1,20E-13	-0,067	4,00E-15	Urea cycle; Arginine and Proline Metabolism	No
Homocitrulline	6859	-0,289	9,70E-132	-0,049	2,20E-08	-0,051	4,70E-09	Urea cycle; Arginine and Proline Metabolism	No
Proline	7081	-0,107	1,30E-19	-0,046	9,20E-09	-0,048	3,90E-09	Urea cycle; Arginine and Proline Metabolism	Yes
Pro-hydroxy-pro	7079	-0,155	1,90E-39	-0,045	3,20E-08	-0,047	5,20E-09	Urea cycle; Arginine and Proline Metabolism	Yes
N-delta-acetylornithine	7078	-0,171	9,00E-48	-0,044	6,60E-08	-0,047	7,70E-09	Urea cycle; Arginine and Proline Metabolism	No
Urea	7082	-0,246	3,00E-98	-0,04	1,70E-06	-0,046	3,60E-08	Urea cycle; Arginine and Proline Metabolism	No

N2,N5-diacetylornithine	6443	-0,231	7,90E-79	-0,042	1,20E-06	-0,043	9,40E-07	Urea cycle; Arginine and Proline Metabolism	No
Trans-4-hydroxyproline	7081	-0,087	1,80E-13	-0,039	1,50E-06	-0,043	1,10E-07	Urea cycle; Arginine and Proline Metabolism	No
Citrulline	7082	-0,195	6,10E-62	-0,038	4,60E-06	-0,037	7,90E-06	Urea cycle; Arginine and Proline Metabolism	No
**Carbohydrates**									
Erythronate*	7079	-0,372	1,20E-230	-0,098	3,60E-29	-0,101	2,00E-30	Aminosugar Metabolism	No
N-acetylneuraminate	7073	-0,194	5,60E-61	-0,039	2,40E-06	-0,045	8,20E-08	Aminosugar Metabolism	No
Mannitol/sorbitol	7070	-0,172	2,70E-48	-0,04	1,20E-06	-0,042	3,10E-07	Fructose, Mannose and Galactose Metabolism	No
Arabonate/xylonate	7063	-0,284	3,70E-131	-0,073	5,70E-18	-0,071	3,30E-17	Pentose Metabolism	No
Arabitol/xylitol	7078	-0,306	5,30E-153	-0,065	3,50E-14	-0,066	2,20E-14	Pentose Metabolism	No
Ribonate	6999	-0,15	1,40E-36	-0,035	2,50E-05	-0,035	2,10E-05	Pentose Metabolism	No
**Cofactors and Vitamins**									
Gulonate*	6162	-0,269	9,10E-103	-0,073	4,70E-16	-0,074	9,20E-17	Ascorbate and Aldarate Metabolism	No
Quinolinate	6836	-0,284	2,00E-127	-0,044	6,20E-07	-0,044	8,40E-07	Nicotinate and Nicotinamide Metabolism	No
**Lipids**									
11beta-hydroxyetiocholanolone glucuronide*	4891	-0,204	2,90E-47	-0,045	3,60E-06	-0,05	4,00E-07	Androgenic Steroids	Yes
3-decenoylcarnitine	5395	-0,217	2,90E-58	-0,043	5,20E-06	-0,042	9,20E-06	Fatty Acid Metabolism (Acyl Carnitine, Monounsaturated)	Yes
Cis-3,4-methyleneheptanoylglycine	6825	-0,161	5,20E-41	-0,034	3,80E-05	-0,038	4,80E-06	Fatty Acid Metabolism (Acyl Glycine)	Yes

Propionylglycine	3960	-0,119	4,90E-14	-0,048	1,70E-05	-0,049	1,30E-05	Fatty Acid Metabolism (also BCAA Metabolism)	Yes
2-methylmalonylcarnitine (C4-DC)	5827	-0,235	8,00E-74	-0,038	2,20E-05	-0,042	3,10E-06	Fatty Acid Metabolism (also BCAA Metabolism)	Yes
Maleate	6952	-0,195	1,00E-60	-0,05	1,80E-09	-0,045	5,60E-08	Fatty Acid, Dicarboxylate	No
Myo-inositol	7076	-0,201	1,10E-65	-0,035	2,20E-05	-0,036	1,40E-05	Inositol Metabolism	No
3-hydroxy-3-methylglutarate	7070	-0,301	1,10E-147	-0,071	8,20E-17	-0,062	4,10E-13	Mevalonate Metabolism	No
Choline	7081	-0,186	3,00E-56	-0,051	4,80E-10	-0,045	4,60E-08	Phospholipid Metabolism	No
**Nucleotides**									
N1-methylinosine	6994	-0,38	1,60E-238	-0,088	5,50E-23	-0,091	2,80E-24	Purine Metabolism, (Hypo)Xanthine/Inosine containing	No
N6-carbamoylthreonyladenosine	6861	-0,386	2,30E-242	-0,087	6,30E-22	-0,097	1,20E-26	Purine Metabolism, Adenine containing	No
N6-succinyladenosine	3784	-0,337	4,40E-101	-0,068	3,10E-09	-0,069	2,30E-09	Purine Metabolism, Adenine containing	No
N1-methyladenosine	7082	-0,286	1,60E-133	-0,06	1,40E-12	-0,066	8,90E-15	Purine Metabolism, Adenine containing	No
N2,N2-dimethylguanosine	7061	-0,377	7,70E-238	-0,088	2,60E-23	-0,091	1,60E-24	Purine Metabolism, Guanine containing	No
7-methylguanine	7082	-0,275	8,50E-123	-0,049	5,90E-09	-0,052	7,50E-10	Purine Metabolism, Guanine containing	No
Orotidine	6601	-0,25	2,10E-94	-0,053	8,80E-10	-0,053	1,80E-09	Pyrimidine Metabolism, Orotate containing	No
Orotate	7010	-0,175	1,40E-49	-0,04	1,10E-06	-0,035	2,40E-05	Pyrimidine Metabolism, Orotate containing	No
Pseudouridine	7079	-0,445	0,00E+00	-0,101	2,70E-27	-0,112	4,40E-33	Pyrimidine Metabolism, Uracil containing	No

3-(3-amino-3-carboxypropyl)uridine*	6366	-0,388	5,20E-228	-0,081	6,60E-18	-0,09	2,00E-21	Pyrimidine Metabolism, Uracil containing	No
5,6-dihydrouridine	7074	-0,341	1,30E-192	-0,067	1,00E-14	-0,076	6,50E-18	Pyrimidine Metabolism, Uracil containing	No
5-methyluridine (ribothymidine)	7082	-0,134	6,80E-30	-0,033	4,60E-05	-0,038	3,10E-06	Pyrimidine Metabolism, Uracil containing	Yes
**Partially characterized molecules**									
Glutamine_degradant*	7060	-0,222	7,30E-80	-0,064	1,40E-14	-0,071	2,20E-17	Partially Characterized Molecules	Yes
Glutamine conjugate of C6H10O2 (1)*	6860	-0,21	3,60E-69	-0,05	1,90E-09	-0,047	3,60E-08	Partially Characterized Molecules	No
Pentose acid*	6808	-0,246	1,10E-94	-0,059	3,20E-12	-0,059	4,70E-12	Partially Characterized Molecules	No
**Peptides**									
N,N-dimethyl-pro-pro	6993	-0,372	5,10E-228	-0,072	8,90E-16	-0,083	2,60E-20	Modified Peptides	No
Pyroglutamylvaline	6398	-0,202	7,70E-60	-0,054	3,80E-10	-0,051	2,60E-09	Modified Peptides	Yes
**Xenobiotics**									
3-methoxycatechol sulfate (2)	5379	-0,185	2,00E-42	-0,046	5,80E-07	-0,044	1,90E-06	Benzoate Metabolism	Yes
4-hydroxyhippurate	7060	-0,191	3,10E-59	-0,046	3,10E-08	-0,043	1,50E-07	Benzoate Metabolism	No
4-methylcatechol sulfate	7076	-0,164	1,10E-43	-0,044	5,40E-08	-0,043	1,40E-07	Benzoate Metabolism	No
3-methyl catechol sulfate (1)	7065	-0,209	3,00E-70	-0,035	2,10E-05	-0,04	2,10E-06	Benzoate Metabolism	Yes
3-methoxycatechol sulfate (1)	6318	-0,174	4,00E-44	-0,04	3,40E-06	-0,039	5,50E-06	Benzoate Metabolism	Yes
4-ethylphenylsulfate	7082	-0,1	2,80E-17	-0,033	4,80E-05	-0,038	3,10E-06	Benzoate Metabolism	No
O-sulfo-L-tyrosine	7082	-0,288	1,70E-135	-0,063	8,90E-14	-0,077	5,10E-19	Chemical	No
6-hydroxyindole sulfate	7070	-0,213	4,40E-73	-0,041	5,80E-07	-0,045	5,50E-08	Chemical	No
Sulfate*	7082	-0,268	1,50E-116	-0,035	5,00E-05	-0,04	2,90E-06	Chemical	No
Hydroquinone sulfate	6400	-0,173	2,60E-44	-0,035	4,10E-05	-0,035	4,70E-05	Drug - Topical Agents	No
Erythritol	7074	-0,385	5,10E-248	-0,103	1,10E-31	-0,104	2,80E-31	Food Component/Plant	No
Gluconate	7048	-0,215	7,70E-75	-0,066	1,80E-15	-0,064	2,60E-14	Food Component/Plant	No

Mannonate*	7073	-0,146	5,30E-35	-0,043	1,10E-07	-0,051	1,80E-09	Food Component/Plant	No
(S)-a-amino-omega-caprolactam	7007	-0,296	1,30E-141	-0,043	7,60E-07	-0,05	1,00E-08	Food Component/Plant	Yes
2,3-dihydroxyisovalerate	6998	-0,206	3,80E-68	-0,05	1,80E-09	-0,048	6,80E-09	Food Component/Plant	Yes
N-(2-furoyl)glycine	5025	-0,235	5,00E-64	-0,044	9,10E-06	-0,042	2,40E-05	Food Component/Plant	Yes
2-acetamidophenol sulfate	5939	-0,153	2,90E-32	-0,043	9,70E-07	-0,042	3,60E-06	Food Component/Plant	Yes
Cinnamoylglycine	6649	-0,181	3,20E-50	-0,042	7,30E-07	-0,039	7,40E-06	Food Component/Plant	No
2-aminophenol sulfate	7066	-0,147	2,80E-35	-0,039	2,20E-06	-0,036	1,10E-05	Food Component/Plant	Yes

Results are based on linear regression. Unadjusted, adjusted for eGFR at baseline as a covariate (Model 1), adjusted for eGFR at baseline, age, BMI, smoking, fasting glucose, total triglycerides and systolic blood pressure (Model 2).
